# North Pacific meridional mode has larger impacts on El Niño evolution than the March Madden-Julian Oscillation

**DOI:** 10.1126/sciadv.adv8621

**Published:** 2025-09-10

**Authors:** Yu Liang, Shang-Ping Xie, Alexey Fedorov, Stephen G. Yeager

**Affiliations:** ^1^ Scripps Institution of Oceanography, University of California San Diego, La Jolla, CA, USA.; ^2^ Department of Earth and Planetary Sciences, Yale University, New Haven, CT, USA.; ^3^ LOCEAN/IPSL, Sorbonne University, Paris, France.; ^4^ NSF National Center for Atmospheric Research, Boulder, CO, USA.

## Abstract

The El Niño–Southern Oscillation (ENSO) is a key driver of global climate variability. Early-season westerly wind bursts (WWBs) have long been suggested to be important for ENSO evolution and diversity, with the Madden-Julian Oscillation (MJO) among the main sources of WWBs. However, MJO’s contribution to ENSO evolution has been difficult to quantify. Here, using an ensemble hindcast approach specifically designed to isolate internal atmospheric variability, we evaluate the influences of March MJO on subsequent ENSO development. Our results show that the March MJO, under favorable background conditions, by itself has limited impacts on ENSO due to weak equatorial air-sea coupling in spring. In comparison, the North Pacific Oscillation–induced meridional mode exerts a more sustained influence on ENSO evolution. A cyclonic circulation anomaly over Hawaii, associated with the Pacific-North American pattern, also plays a role. These findings suggest that March MJO activity alone may not be a reliable predictor for ENSO evolution, but underscore the importance of North Pacific atmospheric variability.

## Teaser

North Pacific atmospheric variability plays a larger role than March MJO activity in driving El Niño evolution.

## INTRODUCTION

The El Niño–Southern Oscillation (ENSO) is the dominant mode of interannual climate variability, exerting huge impacts on global climate, ecosystems and societies (*1*, *2*). Extreme El Niño events, such as the 1997–1998 El Niño, can cause severe consequences, including droughts in Australia and Indonesia (*3*, *4*), widespread wildfires in Indonesia (*5*), and record-breaking floods in the Yangtze River basin (*6*–*8*). Given these profound impacts, improving our understanding of mechanisms that govern the development and intensity of extreme El Niño events is critical for enhancing ENSO predictions and societal preparedness.

Recharged ocean heat content (OHC) and westerly wind bursts (WWBs) during boreal winter and spring have been suggested as key elements in extreme El Niño events (*9*–*14*). While OHC is slow varying, WWBs, episodes of strong westerly wind anomalies over the western-central equatorial Pacific, are much more variable and unpredictable, complicating El Niño prediction (*15*). Among various sources of WWBs, the Madden-Julian Oscillation (MJO), the dominant intraseasonal mode in the tropical atmosphere (*16*, *17*), plays a critical role (*18*–*21*). For instance, an intense WWB in March 1997, which helped trigger the 1997–1998 extreme El Niño (*22*), was linked to a strong MJO event (*23*) (also see fig. S1A). Tropical cyclones embedded within the MJO can further amplify WWBs (*18*). Although enhanced MJO activity over the western equatorial Pacific during boreal spring and early summer often precedes El Niño development later in the year (*24*, *25*), the precise contribution of the MJO remains a subject of debate (*26*). The challenge in attributing the MJO’s influence stems from its coexistence with seasonal westerly wind anomalies linked to the eastward expansion of the warm pool (*25*, *27*), complicating the separation of MJO-driven effects from those of the background sea surface temperature (SST) variations (*27*, *28*).

Other climate modes further complicate the attribution. In particular, the Pacific Meridional Mode (PMM) has emerged as a critical factor (*29*). Originating in the subtropical northeastern Pacific, the PMM propagates southwestward toward the equator via the wind-evaporation-SST feedback (*30*), inducing westerly wind anomalies over the western-central equatorial Pacific and contributing to El Niño development (*31*). The co-occurrence of a strong MJO and PMM activity in March 1997 illustrates the complexity in distinguishing their respective roles. There has also been debate about whether the PMM is an independent mode from the Central Pacific type of El Niño (*32*). However, studies have suggested that the North Pacific Oscillation (NPO) (*33*), an internal atmospheric mode in mid-latitudes, can trigger the PMM during boreal winter (*29*).

In this study, we present an approach using an ocean-atmosphere coupled model ensemble hindcast, specifically designed to isolate and quantify the influences of different atmospheric modes, including the MJO and NPO, on ENSO evolution. By leveraging the ocean’s long memory (several months to a year) (*34*, *35*) compared to the atmosphere’s [about 3 weeks for the MJO (*36*), and 1 week for mid-latitude weather systems (*37*)], we effectively isolate the atmospheric modes from the early-stage atmospheric ensemble spread, when changes in the ocean state (e.g., SST) are minimal. This approach allows us to quantify the distinct contributions of atmospheric modes to ENSO evolution, addressing the challenge posed by the inherently coupled nature of air-sea interactions. By better understanding the role of the early-season MJO and NPO, we can improve ENSO prediction, in particular in boreal spring, a period characterized by reduced forecast skill (*38*, *39*).

## RESULTS

### March MJO

**Fig. 1.**
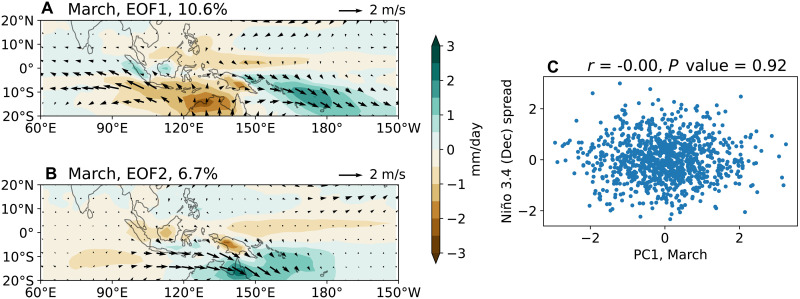
The first two EOFs of the March MJO. (**A**) EOF1 and (**B**) EOF2, derived from the March ensemble spread of precipitation and surface winds in the tropical Indo-Pacific region (60°E to 150°W, 20°S to 20°N). They explain 10.6 and 6.7% of the total variance, respectively. We focus on EOF1 as it generates westerly wind anomalies over the western equatorial Pacific, potentially affecting El Niño. (**C**) Scatter plot showing the relationship between the principal component of EOF1 (PC1) and Niño 3.4 spread in the following December, illustrating insignificant correlation between the two.

We analyze hindcasts initialized on February 1st from the seasonal-to-multiyear large ensemble (SMYLE), spanning 1970–2019 and produced using the Community Earth System Model version 2 (CESM2) (*40*) (see Materials and Methods). Empirical orthogonal function (EOF) analysis is performed on the ensemble spread of March monthly precipitation and surface winds in the tropical Indo-Pacific region (see Materials and Methods). The two leading EOFs are shown in Fig. 1. EOF1 features a dipole pattern characterized by westerly wind anomalies over the western equatorial Pacific, which favors WWB occurrence (*18*), whereas EOF2 primarily displays wind anomalies over the Maritime Continent. Their horizontal structures resemble the intraseasonal MJO simulated by CESM2 (fig. S2, G and H). Given that EOF2 has minimal influences on equatorial Pacific Ocean processes, we focus on EOF1 hereafter.

As the MJO exhibits a spectral peak at intraseasonal periods of 20 to 100 days (*41*), considerable MJO variability remains even after monthly averaging; the EOF1 and EOF2 can therefore be used to represent the MJO. However, in addition to the intraseasonal MJO signal, EOF1 also captures a recently identified intrinsic low-frequency atmospheric mode. This mode structurally resembles the MJO but has a nearly white spectrum at periods beyond 100 days (*42*), previously described as the MJO’s “low-frequency tail” (*43*). We estimate that the intraseasonal MJO contributes ~46% of EOF1 variance, whereas this low-frequency MJO-like mode accounts for ~54% (See Materials and Methods).

We examine the impact of EOF1 on subsequent ENSO evolution, which reflects the combined effects of both intraseasonal MJO and the low-frequency MJO-like mode. This approach aligns with operational MJO monitoring practice, such as the Real-time Multivariate MJO index (*44*), where westerly wind anomalies in the western equatorial Pacific, triggered by MJO-like events, inherently blend intraseasonal MJO and the low-frequency MJO-like mode signals. Henceforth, we refer to EOF1 simply as “MJO,” while noting that it encompasses both components. Later, we separately assess the distinct impacts of the intraseasonal MJO and its low-frequency counterpart.

To assess whether the March MJO significantly influences subsequent El Niño development, we compute the correlation between PC1 and Niño 3.4 spread in the following December. The correlation is nearly zero (Fig. 1C), indicating that the March MJO has little effect on wintertime El Niño amplitude.

**Fig. 2.**
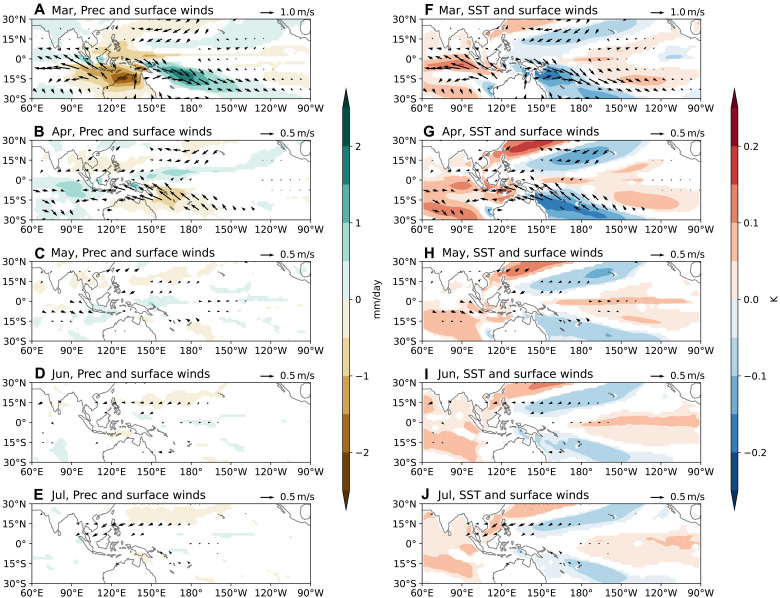
The ocean-atmosphere response to the March MJO. (**A** to **E**) Ensemble spread of precipitation (color shading) and surface winds (arrows) from March to July, regressed onto the PC1 of EOF1 in Fig. 1A. (**F** to **J**) Same as the left panels, but with SSTs shown by color shading. Note that the scale for wind vectors differs in (A) and (F) from the other panels. Hereafter, only regressions statistically significant at the 95% confidence level are shown.

**Fig. 3.**
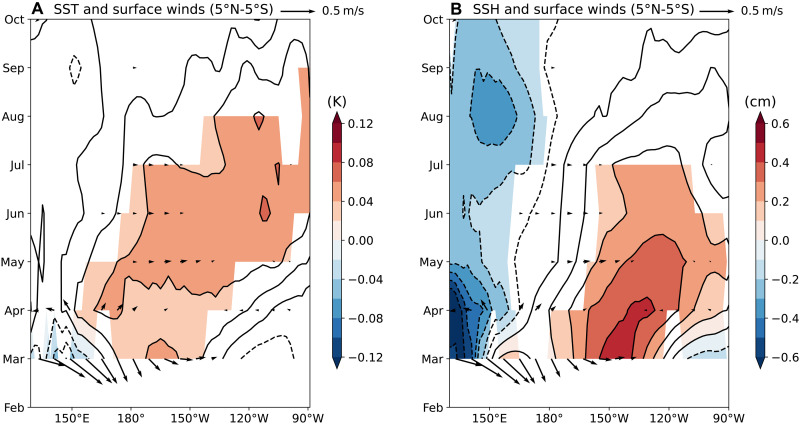
Equatorial oceanic and atmospheric response to the March MJO. (**A**) Ensemble spread of SST (contours) and surface wind vectors, averaged between 5°N to 5°S, regressed onto the PC1 of March MJO. Color shading denotes SST correlations statistically significant at the 95% confidence level. (**B**) Same as (A), but contours and color shading represent equatorially averaged sea surface height (SSH) regressed onto the PC1 of March MJO.

Further analysis reveals that westerly wind anomalies associated with the March MJO induce cooling in the South Pacific Convergence Zone, where climatological winds are northwesterly (Fig. 2F). These westerly wind anomalies push the eastern edge of the warm pool eastward through zonal advection, leading to warm SST anomalies in the western equatorial Pacific by April (Fig. 2G). These warm SST anomalies enhance precipitation and generate additional weak westerly wind anomalies (Fig. 2B), suggesting positive feedback. Concurrently, the March MJO generates a downwelling oceanic Kelvin wave (Fig. 3B), resulting in warm SST anomalies in the central-eastern equatorial Pacific by May (Fig. 2H). However, these equatorial SST anomalies are only weakly coupled with precipitation and surface winds (Figs. 2, C to E and H to J, and 3A), and the signals dissipate by August. As a result, the March MJO fails to produce significant warming in December.

We further examine the atmosphere-ocean responses to the intraseasonal and low-frequency components of the March MJO (see Materials and Methods). Following the intraseasonal westerly wind anomalies over the western equatorial Pacific in March, relatively strong easterly wind anomalies develop in April (fig. S5B), effectively neutralizing the equatorial oceanic response by May (fig. S5H). In contrast, following the low-frequency westerly wind anomalies in March, equatorial westerly wind anomalies persist in April, albeit with weaker amplitude, due to the eastward expansion of the warm pool (fig. S6G). Comparing Fig. 2 (H to J) and fig. S6 (H to J), we conclude that the net downwelling oceanic Kelvin waves observed from May to July in Fig. 2 are mainly driven by the low-frequency MJO-like mode in March.

### March NPO and PNA

**Fig. 4.**
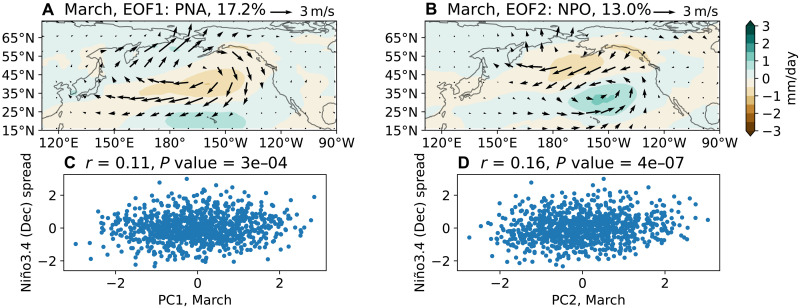
The first two EOFs of North Pacific atmospheric variability in March. (**A**) EOF1, representing the Pacific-North American pattern. (**B**) EOF2, capturing the NPO variability. (**C** and **D**) Correlation between PCs of these modes and Niño 3.4 spread in the following December, illustrating their respective contributions to ENSO development.

The NPO is widely recognized as a key driver of the PMM, which can generate westerly wind anomalies in the western equatorial Pacific and favor El Niño development (*29*, *31*, *45*). To isolate the March NPO, we perform an EOF analysis on the ensemble spread of monthly precipitation and surface winds over the North Pacific (see Materials and Methods). EOF1 corresponds to the Pacific-North American (PNA) pattern (*46*), while EOF2 captures the NPO variability (Fig. 4, A and B). Both modes exhibit positive correlations with El Niño amplitude in the subsequent winter (Fig. 4, C and D). Random sampling indicates that at least 200 members are required to robustly detect this correlation. That the negative phase of PNA in March favors El Niño growth aligns with previous observational evidence linking the March Aleutian Low intensity with following El Niño amplitudes (*47*); however, our analysis suggests that the strength of this connection may have been overestimated previously.

**Fig. 5.**
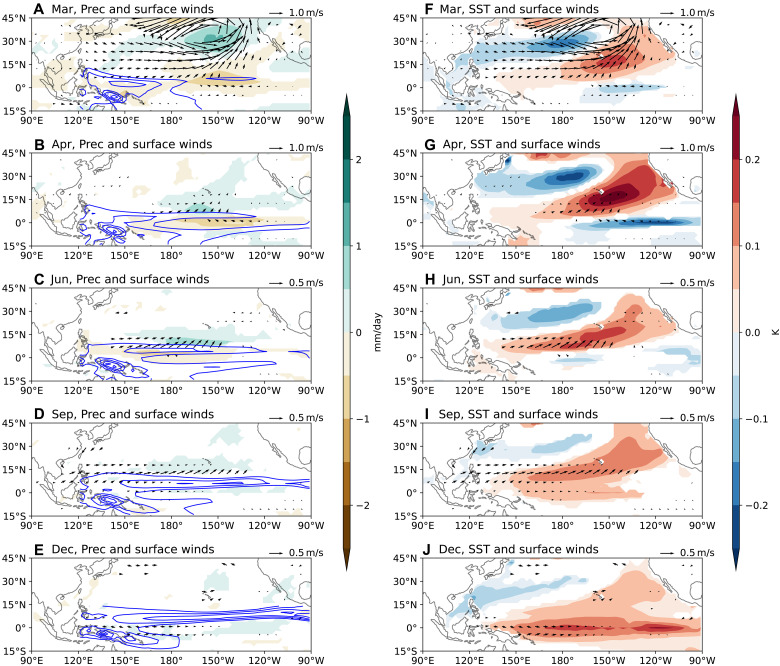
Ocean-atmosphere response to the March NPO. (**A** to **E**) Ensemble spread of precipitation (color shading) and surface winds (arrows) regressed onto the PC2 of EOF2 in Fig. 4B. (**F** to **J**) Same as the left panels, but with SSTs shown by color shading. Blue contours in the left panels depict monthly climatological precipitation, with a contour interval of 3 mm/day starting at 6 mm/day. Note that the scale of surface wind vectors varies across panels.

We then examine the impact of the March NPO on ENSO evolution by regressing the ensemble spread fields onto PC2. As illustrated in Fig. 5F, our method effectively isolates the NPO with minimal influence from the equatorial ocean: SST anomalies in the central equatorial Pacific (i.e., 150°E to 150°W, 5°N to 5°S), a region suggested to be important for PMM excitation (*32*), are weak in March. The cold SST anomalies in the eastern equatorial Pacific are likely driven by local easterly wind anomalies, themselves a direct response to the warm PMM SST anomalies induced by the NPO. Additional atmospheric experiments (see Materials and Methods) confirm that such weak SST anomalies cannot excite the extratropical circulation anomalies observed in Fig. 5A (fig. S7A).

The positive NPO is associated with anomalous southwesterlies in the trade wind region, which reduce evaporation and warm SSTs (Fig. 5F). These subtropical warm SST anomalies peak in April (Fig. 5G) and subsequently interact with the intertropical convergence zone (ITCZ), displacing it northward. Meanwhile, the NPO-induced PMM propagates southwestward toward the equator. By August-September, anomalous westerlies emerge on the equator through the summer deep convection mechanism (*48*, *49*), initiating the Bjerknes feedback (*50*), with equatorial SST anomalies reaching approximately 0.2 K by December (Fig. 5J). This evolution is consistent with the seasonal footprinting mechanism described in previous studies (*51*–*53*).

Further examination of ocean sea surface height response reveals that as the PMM propagates westward, it also generates forced downwelling oceanic Rossby waves (figs. S8 and S9), recharging the upper OHC (*54*). These Rossby waves arrive at the western boundary around August, where they reflect to become eastward-propagating Kelvin waves likely by September. These reflected Kelvin waves, lagging those generated by the equatorial westerly wind anomalies in August, further contribute to El Niño development (fig. S9).

Unlike the transient equatorial warming caused by the March MJO, which dissipates within 2 to 3 months, the NPO-generated PMM persists significantly longer, influencing ENSO evolution throughout the year. The mechanisms sustaining this persistence are examined in the next subsection.

**Fig. 6.**
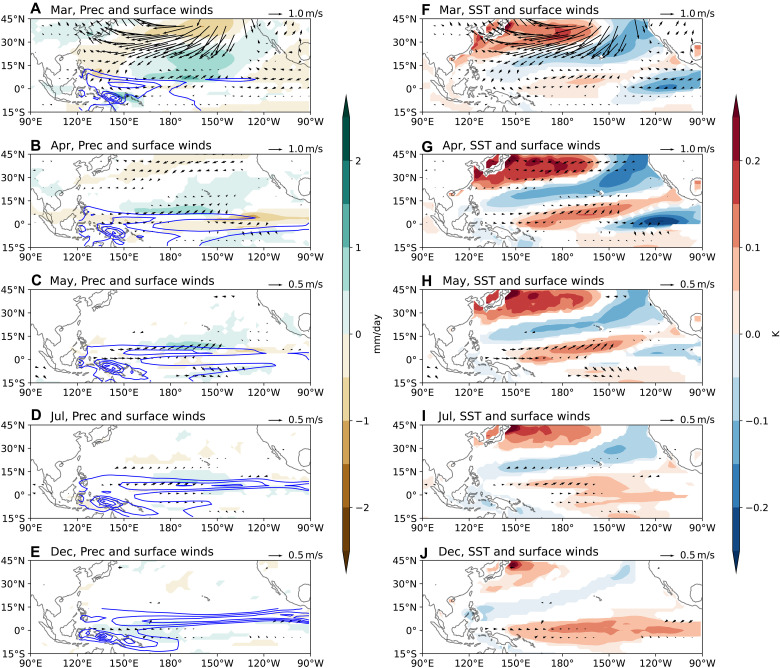
Ocean-atmosphere response to the March PNA. (**A** to **E**) Ensemble spread of precipitation (color shading) and surface winds (arrows) regressed onto the PC1 of EOF1 in Fig. 4A. (**F** to **J**) Same as the left panels, but with SSTs shown by color shading. Blue contours in the left panels depict monthly climatological precipitation, with a contour interval of 3 mm/day starting at 6 mm/day. We have chosen different months from Fig. 5 as the temporal evolution of the ocean-atmosphere response is different. Note that the meridional mode-like SST anomalies are primarily generated by the cyclonic circulation south of the Aleutian Low, and the scale of surface wind vectors varies across panels.

We also compare the ocean-atmosphere response to the PNA with that to the NPO. Notably, the cyclonic circulation south of the weakened Aleutian Low, part of the PNA structure (fig. S10), generates meridional mode-like SST anomalies by reducing evaporation (Fig. 6, F and G). Located further south than the NPO’s southern lobe, this cyclonic circulation directly induces westerly wind anomalies on the equator (Fig. 6F), and the resulting meridional mode–like SST anomalies are orthogonal to those associated with the NPO, with a pattern correlation close to zero in April. The cold SST anomalies in the far eastern equatorial Pacific are also directly forced by the PNA-related circulation anomalies; these SST anomalies first amplify in April and then gradually decay.

By interacting with the ITCZ (Fig. 6, B to D), the meridional mode–like warm SST anomalies generate additional westerly wind anomalies both along and north of the equator. These westerly wind anomalies drive downwelling oceanic Kelvin waves (figs. S11 and S12), causing significant warm SST anomalies in the central equatorial Pacific by July (Fig. 6I), approximately 2 months earlier than those induced by the March NPO. The subsequent Bjerknes feedback further amplifies these SST anomalies, reaching ~0.15 K by December (Fig. 6J).

Unlike the NPO case, the meridional mode–like SST anomalies associated with the PNA remain largely stationary, and no clear westward propagating downwelling Rossby waves are observed (figs. S11 and S12B). This absence of Rossby waves suggests that the trade wind charging mechanism does not play a role in this case.

### Mechanisms maintaining the NPO-induced PMM

**Fig. 7.**
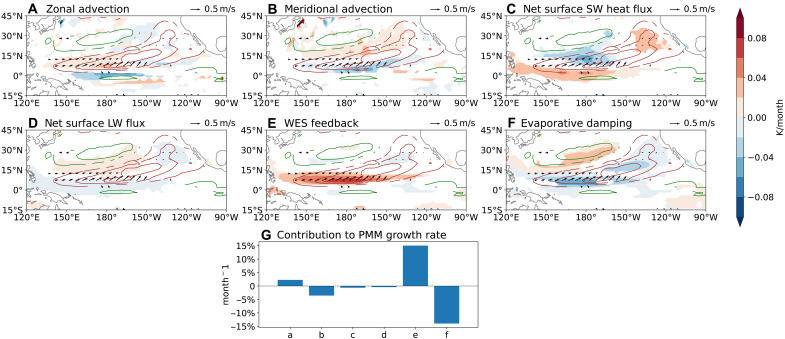
Heat budget terms for the ocean’s mixed layer (upper 50 m) in June. (**A**) Zonal advection, (**B**) meridional advection, (**C**) net surface shortwave heat flux, (**D**) net surface longwave heat flux, (**E**) wind-evaporation-SST (WES) feedback, and (**F**) evaporative damping associated with the NPO-induced PMM (see Materials and Methods). Vertical advection and sensible heat flux terms are very small. Positive heat flux indicates energy gain by the ocean. Brown and green contours indicate positive and negative SST anomalies in Fig. 5H, respectively. Arrows represent the associated surface wind anomalies. (**G**) Normalized contribution of each term from (A) to (F) to the PMM maintenance between April and August (see Materials and Methods).

The long persistence of PMM is crucial for the March NPO to influence the El Niño amplitude in the following December. While the WES feedback is recognized important in the PMM dynamics (*29*, *55*), its relative role compared to other physical processes has not been fully quantified. To address this gap, we conduct a monthly mixed-layer heat budget analysis, explicitly accounting for the nonstationary evolution of the NPO-induced PMM from April through August (see Materials and Methods). In particular, we decompose the latent heat flux term into WES feedback and evaporative damping components, a refinement not undertaken in prior studies (*55*). As shown in Fig. 7, the WES feedback emerges as the dominant driver of PMM maintenance, peaking southwest of the PMM center where southwesterly wind anomalies are the strongest. Its effect is largely counteracted by evaporative damping, which arises from increased SST and the resulting larger air-sea specific humidity difference (*56*). Without the decomposition, the role of the WES feedback would be obscured.

Other heat flux terms play secondary roles. Net surface longwave heat flux is nearly zero. Net surface shortwave heat flux exhibits distinct spatial patterns: it is positive along the equator and in the subtropical northeastern Pacific, but negative north of the ICTZ, associated with the northward displaced ITCZ and positive low cloud-SST feedback in the subtropical northeastern Pacific (*56*). Despite these local variations, the overall contribution of the shortwave flux to the PMM maintenance remains minor. However, our local-scale heat budget may not fully capture potentially important nonlocal effects, such as the low cloud-SST feedback along the PMM’s southwestward propagation pathway (*56*, *57*). In addition, zonal advection is found to support PMM maintenance, while meridional advection acts to damp it, two dynamical processes often overlooked in previous studies.

Our analysis also sheds light on the southwestward propagation mechanism of the PMM. The WES feedback appears to be the primary driver of this propagation. However, as the associated westerly wind anomalies are located north of the ITCZ center, they prevent the PMM from reaching the equator before August–September. The net surface shortwave heat flux also favors equatorward migration of the PMM by warming SSTs in the equatorial band, but this warming is largely canceled by cooling from zonal advection. As a result, equatorial SST anomalies only emerge once off-equatorial PMM SST anomalies induce equatorial westerly wind anomalies.

### A case study: The MJO variability in March 1997

The March MJO fails to influence the El Niño amplitude in the following December, partly due to its relatively weak equatorial westerly wind anomalies (Fig. 1A and Table 1). In observations, only ~40% of MJO events in boreal winter-spring generate WWBs, which typically occur under favorable background conditions, such as an eastward-expanded warm pool and associated seasonal westerly anomalies (*18*–*20*). These conditions enhance MJO convection and westerly wind anomalies on the equator.

**Table 1.** Surface zonal wind anomalies (m/s) averaged over the region 130°E to 180°, 5°S to 5°N for various cases. The first three cases are model-simulated: (i) the average March MJO (see Fig. 1A), (ii) the MJO in March 1997 (see Fig. 8A), and (iii) the 50-member ensemble average in March 1997 associated with a North Pacific meridional mode (NPMM). The final case (iv) represents the observed wind anomalies in March 1997.

Average March MJO	March 1997, MJO	March 1997, composite NPMM	March 1997, observations
0.33 m/s	0.78 m/s	1.18 m/s	4.28 m/s

**Fig. 8.**
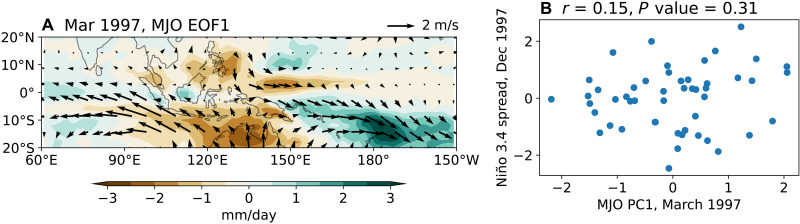
Simulated MJO in March 1997, and its impacts on the following winter El Niño. (**A** and **B**) As in Fig. 1 (A and C), but with the EOF analysis performed only on the 50-member ensemble spread in March 1997.

In March 1997, a warm meridional mode–like SST anomaly provided such favorable conditions (fig. S13). As a result, the March 1997 MJO, derived from the 50-member ensemble spread (see Materials and Methods), exhibits equatorial westerly wind anomalies more than twice as strong as the average March MJO (Fig. 8A versus Fig. 1A, and Table 1). Despite this enhanced forcing, the correlation between the MJO amplitude and the Niño 3.4 spread in December 1997 remains statistically insignificant. To test robustness, we expand the ensemble to 250 members by including years in which the 20-member ensemble-mean SST anomalies exceed 0.3 K in the western-central equatorial Pacific (150°E to 170°W, 5°N to 5°S) in March (*18*) and 0.5 K in the Niño 3.4 region in the following December. Even with this larger sample, the correlation remains insignificant (fig. S14).

The simulated March 1997 MJO wind anomalies (Fig. 8A) closely resemble the observed wind anomalies (fig. S1B). However, while Fedorov *et al.* (*11*) found that imposing the observed March 1997 wind stress anomalies substantially increased the Niño3 index (~2 K) by December 1997, our results indicate no significant enhancement in El Niño amplitude in response to the March MJO forcing. We attribute this discrepancy to differences in the wind anomaly strength. The observed westerly wind anomalies in March 1997 were about 4.3 m/s over 130°E to 180°, 5°S to 5°N (Table 1), whereas the simulated March 1997 MJO generates anomalies of only about 0.78 m/s (one SD; Fig. 8A and Table 1). Even a particularly strong simulated MJO (two SDs, ~5% chance; Fig. 8B) yields anomalies around 1.6 m/s, about 40% of the observed strength.

Several factors explain this difference: First, the observed March 1997 WWB had multiple contributors beyond the MJO, including the preexisting North Pacific Meridional Mode (NPMM; fig. S13 and Table 1) and embedded tropical cyclones, both of which are known to enhance WWBs substantially (*18*, *58*). Second, the simulated intraseasonal MJO exhibits weaker equatorial westerly anomalies (about 60% of observed values; fig. S2), and westerly wind anomalies of the MJO defined in this study, which includes an MJO-like low-frequency mode (*42*), may also be underrepresented. Last, we note that in SMYLE, the westerly anomalies induced by the March MJO are partially offset by intraseasonal easterly anomalies in April (fig. S5B and Fig. 2B), a constraint absent in the idealized experiments of Fedorov *et al.* (*11*). These findings do not negate Fedorov *et al.*’s (*11*) results, but necessitate other factors, in addition to the MJO, to boost WWBs (*18*).

## DISCUSSION

Previous studies have highlighted the important role of early-season WWBs in generating extreme El Niño events and shaping ENSO diversity (*11*–*14*, *59*, *60*). Using an ensemble hindcast approach specifically designed to isolate internal atmospheric variability, this study extends the analysis to examine the individual effects of the MJO, NPO, and PNA in March on subsequent ENSO evolution within a consistent ensemble framework. All three atmospheric modes can directly or indirectly generate westerly wind anomalies in the equatorial band. Note that while we have focused on atmospheric modes in the Pacific, other climate modes outside the Pacific could also affect ENSO evolution (*61*, *62*).

We find that while the March MJO generates the strongest westerly wind anomalies on the equator, its influence on ENSO development is limited due to weak equatorial air-sea coupling in subsequent months; note that its impacts on the equatorial ocean are dominated by an MJO-like low-frequency mode (*42*). In contrast, the NPO-induced PMM has stronger impacts on ENSO, driven by longer persistence of the PMM SST anomalies through WES feedback and coupling with the ITCZ. The PNA also contributes to ENSO development via a cyclonic circulation near Hawaii, inducing warm SST anomalies both on the equator and north of the equator. However, correlations are generally low, consistent with the well-known ENSO spring predictability barrier. Nonetheless, these findings suggest that improving the representation of North Pacific atmospheric variability and its interaction with ENSO in physical models could enhance dynamical ENSO predictions.

The limited effect of the March MJO on the following December El Niño amplitude, even under favorable background conditions, is somewhat unexpected. Given that the MJO in December, January, and February typically exhibits westerly wind anomalies farther from the equator and occurs earlier in the year, their individual contributions to subsequent El Niño evolution are also likely small. However, it is still possible that a sequence of convective MJO events from December to March can collectively induce sufficient warming in the central-eastern equatorial Pacific during summer, which is sustained till December. In addition, tropical cyclones embedded in the MJO can greatly strengthen westerly wind anomalies in the equatorial band. This was apparently the case in 2015, when three successive WWBs, occurring in January through March and amplified by tropical cyclones, led to a strong warming in the east that culminated in a strong El Niño event (*12*). Note that CESM2 currently still underestimates the MJO’s equatorial wind variability, which may hinder the ability of the March MJO to affect the December El Niño amplitude. With continued improvements of numerical models, more accurate assessments of the MJO impacts may be possible.

Our findings on the role of the March MJO should not be generalized to later months, such as April or May. During these months, the MJO shifts northward, its westerly wind anomalies on the equator strengthen, and the time proximity to December increases. In this study, by April and May, the equatorial SST spread in the February 1st initialized hindcasts becomes notable, which modifies tropical precipitation and complicates isolating the MJO signal without the SST influence.

Last, we stress that the methodology used in this study is not limited to ENSO but can also be applied to other modes of air-sea interaction, such as the Indian Ocean Dipole (*63*) and Atlantic Niño (*64*). This approach offers a pathway to better understand how atmospheric processes affect the ocean and subsequent ocean-atmosphere coupling.

## MATERIALS AND METHODS

### Dataset

We use the SMYLE set of initialized hindcasts generated with the CESM2 (*40*). CESM2 is configured with a 1°-horizontal resolution ocean model (POP2) and a 1°-horizontal resolution atmosphere model (CAM6-FV). The hindcasts are initialized quarterly from 1970 to 2019, and for this study, we use the February 1st initialization to investigate the impacts of different atmospheric modes in March on subsequent ENSO evolution. The SMYLE consists of 20 ensemble members, each integrated for 2 years. To perform the March 1997 case study, we conducted an additional 30 ensemble member experiments for the 1 February 1997 initialization.

CAM6 control run forced with climatological monthly SST/sea ice from years 401 to 2000 of the CESM2.1 coupled control run (https://cesm.ucar.edu/working-groups/climate/simulations/cam6-picontrol) is used to isolate the PNA as an intrinsic atmospheric mode.

### Isolating atmospheric modes

To isolate different atmospheric modes, we perform EOF analysis on the atmospheric ensemble spread fields for March. During this month, the SST spread across the 20 ensemble members is minimal (about 0z.2 K in the Niño 3.4 region), ensuring that the atmospheric ensemble spread primarily reflects internal atmospheric variability. The ensemble spread is calculated separately for each year’s initialization by subtracting the ensemble mean from each member. With hindcasts spanning from 1970 to 2019, this approach produces a total of 1000 ensemble spread samples.

For isolating the MJO in Fig. 1, EOF analysis is performed on March-mean precipitation, zonal, and meridional surface winds over the region 60°E to 150°W, 20°N to 20°S, following Liang *et al.* (*42*). To isolate the NPO, EOF analysis is performed on the same fields over the region 110°E to 90°W, 15°N to 75°N. Before conducting the EOF analysis, all fields are interpolated onto a 2°-horizontal resolution grid. To quantify the impacts of the atmospheric modes on ENSO evolution, we regress the ensemble spread of SST, precipitation, surface winds, and ocean temperature onto the March principal components (PCs).

We compare the simulated intraseasonal (20- to 100-day bandpass filtered) MJO, PNA, and NPO in March with those in the ERA5 reanalysis. As shown in fig. S2 (E and F), the MJO in SMYLE closely resembles that in the reanalysis (fig. S2, A and B), although its zonal wind variability in the western equatorial Pacific (1°N to 5°S, 130°E to 180°) is approximately 60% of the observed strength. SMYLE also captures the horizontal structures of the PNA and NPO, although they are displaced roughly 8° equatorward compared to ERA5 (fig. S3). This equatorward shift could potentially enhance their influence on ENSO evolution, but the weaker cyclonic circulation compared to ERA5 may counteract this effect.

As discussed in the main text, EOF1 shown in Fig. 1A captures variability associated with both intraseasonal MJO and an MJO-like low-frequency atmospheric mode. To quantify their individual contributions to the EOF1 variability, we first isolate precipitation using a 20- to 100-day bandpass filter for the intraseasonal component and a 100-day lowpass filter for the low-frequency component, and then compute their March monthly means. Next, we calculate the ensemble spread of these filtered March precipitation fields and project this spread onto the precipitation anomalies associated with EOF1. This procedure yields two new PC series: PC1_Intra for the intraseasonal component and PC1_LF for the low-frequency component.

We then compute the correlation coefficients between PC1 and each of these new series, obtaining values of 0.74 for PC1_Intra and 0.80 for PC1_LF (fig. S4). The squares of these correlation coefficients represent the fraction of EOF1 variability explained by each component. Note that the sum of these fractions exceeds unity because PC1_Intra and PC1_LF are partially correlated due to the approximate nature of the 100-day cutoff between intraseasonal and low-frequency variations. By normalizing, we estimate the relative contributions to the EOF1 variability as approximately 46% for the intraseasonal MJO and 54% for the MJO-like low-frequency mode.

### Atmospheric experiments

To validate that the atmospheric ensemble spread in March primarily reflects internal atmospheric variability with minimal influence from the SST spread (cf. Figs. 5F and 6F), we perform additional atmospheric model experiments using CAM6. In the control experiment, CAM6 is forced with perpetual climatological March conditions (i.e., SST and insolation) and integrated for 5 years following a 2-year spin-up. In the first perturbation experiment, the SST anomalies shown in Fig. 5F are added to the SST forcing field, and the model is integrated for 5 years. In the second perturbation experiment, the SST anomalies shown in Fig. 6F are similarly added. The atmospheric responses to the imposed SST anomalies are presented in fig. S7.

### Ocean mixed layer budget analysis

We conduct an ocean mixed layer budget analysis to identify processes that maintain the PMM SST anomalies. The heat budget equation is

$$\begin{array}{c} \begin{array}{c} \partial T^{\prime }/\partial t={\left(-\;u\partial T/\partial x\right)}^{\prime }-{\left(v\partial T/\partial y\right)}^{\prime }-{\left(w\partial T/\partial z\right)}^{\prime }\\ +SW^{\prime }+LW^{\prime }+\text{LH}F^{\prime }+\text{SH}F^{\prime }\; \end{array} \end{array}$$ (1)

where *T* represents ocean temperature; *u*, *v*, and *w* are the zonal, meridional, and vertical current velocities, respectively; and SW and LW indicate net surface shortwave and longwave heat fluxes, respectively. LHF and SHF are latent and sensible turbulent heat fluxes, respectively. Positive heat flux values indicate that the ocean is gaining energy. The prime (′) indicates the ensemble spread, and all terms are averaged over the upper 50 m of the mixed layer.

We further decompose LHF′ into components of WES feedback and evaporative damping, following Yang *et al.* (*56*). The WES feedback is defined as

$$\text{WES}=U^{\prime }/U_{\text{clim}}\text{LH}F_{\text{clim}}$$ (2)

where $U^{\prime }\;$represents the spread of surface wind speed, and $U_{\text{clim}}$ and $\text{LH}F_{\text{clim}}$ are monthly climatological surface wind speed and latent heat flux, respectively. The evaporative damping is computed as the residual

$$\text{EVAP}=\text{LH}F^{\prime }-U^{\prime }/U_{\text{clim}}\text{LH}F_{\text{clim}}$$ (3)

To quantify the contribution of each term on the right-hand side of Eq. 1 to the maintenance of the PMM SST anomalies, we project these terms onto the positive PMM SST anomalies from April to August, before the emergence of warm SST anomalies on the equator (c.f., Fig. 5). Here, we focus exclusively on the warm SST anomalies, as they persist into summer; in comparison, the negative equatorial SST anomalies gradually decay after April. This approach has been previously used in the MJO studies (*65*, *66*). The contribution of each term is calculated as

$$C_{x}=\frac{\left\|x\cdot T^{\prime }\right\|}{\left\|{T^{\prime }}^{2}\right\|}$$ (4)

where *x* is a heat budget term, and ||*y*|| denotes the space-time integral of *y*, defined as

$$\|y\|=\int_{t}\iint_{\text{warm}}\mathrm{ydA}\;\mathrm{dt}$$ (5)

where the spatial integral is over regions where the PMM SST anomalies are positive, and the time integral covers the period from April to August.

### Statistical analysis

The Pearson correlation coefficient (denoted as *r*) is used to measure the correlation between two variables. A two-tailed Student’s *t* test with *P* value of 0.05 is then used to determine whether the correlation/regression coefficients are statistically significant at the 95% confidence level.
