# Supplementary material for: North Pacific meridional mode has larger impacts on El Niño evolution than the March Madden-Julian Oscillation
Source: Sci Adv. 2025 Sep 10;11(37):eadv8621. doi: 10.1126/sciadv.adv8621 (PMC12422190; doi:10.1126/sciadv.adv8621)
Supplement: Supplementary file 1 — Figs. S1 to S14 [file sciadv.adv8621_sm.pdf]

Science Advances
AAAS

Supplementary Materials for

North Pacific meridional mode has larger impacts on El Niño evolution than the March Madden-Julian Oscillation

Yu Liang *et al.*

Corresponding author: Yu Liang, yul257@ucsd.edu

Sci. Adv. **11**, eadv8621 (2025)
DOI: 10.1126/sciadv.adv8621

This PDF file includes:

Figs. S1 to S14

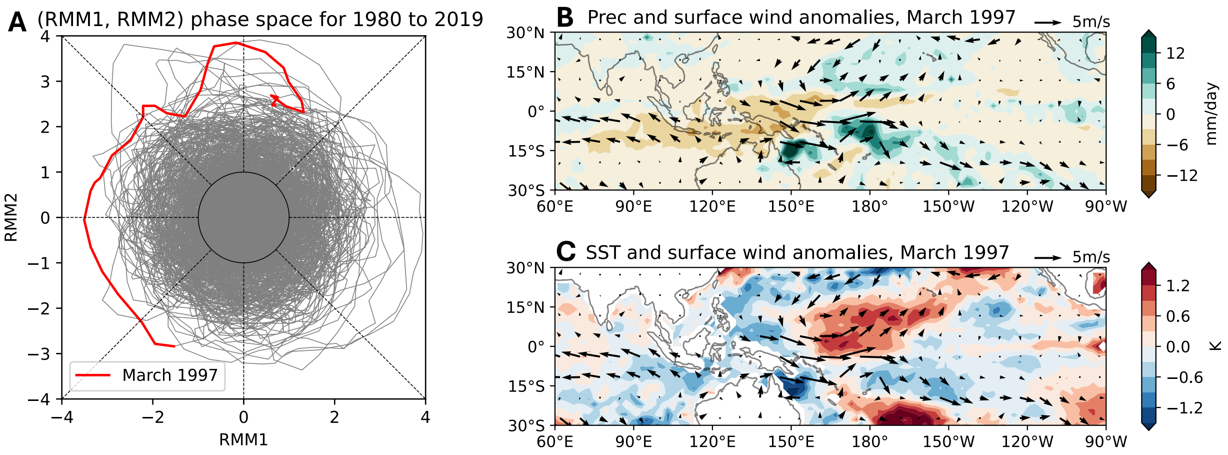

fig. S1. Atmospheric and oceanic conditions in March 1997. (**A**) Phase space diagrams for the MJO from January 1st, 1980 to December 31st, 2019 in gray lines. The red line indicates the MJO condition in March 1997. (**B**) Anomalous precipitation in color shading and surface winds in black arrows in March 1997. (**C**) Anomalous sea surface temperature (SST) in color shading. Anomalies are calculated relative to the period of 1970-2019.

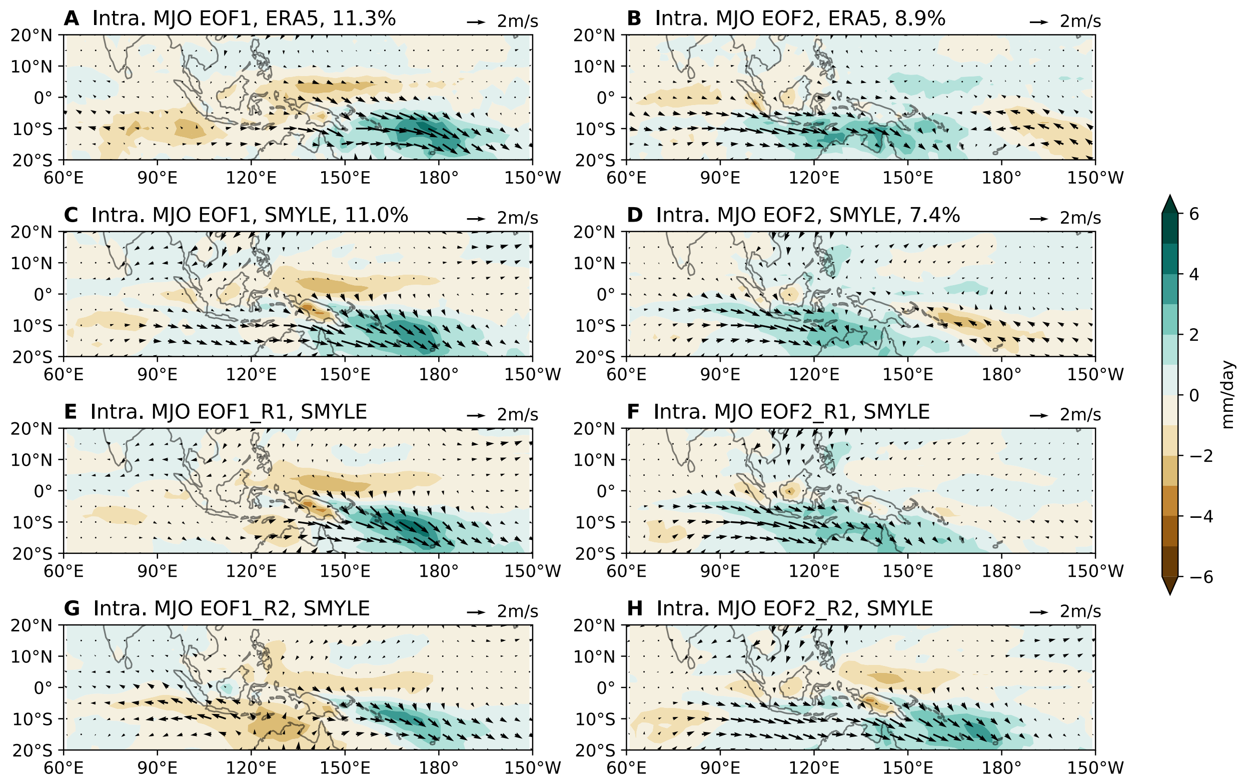

fig. S2. Horizontal structures of the MJO (20-100 day bandpass filtered) in the ERA5 reanalysis and SMYLE during March. (**A**, **B**) The first two EOFs of bandpass filtered precipitation and surface winds in the reanalysis, representing the horizontal structure of the observed MJO. (**C**, **D**) As in (**A**, **B**) but based on the SMYLE data. The numerical percentages in the upper-right corner of panels (**A-D**) represent the proportion of total variance explained by each EOF. (**E**, **F**) Rotated MJO EOF1 and EOF2 in (**C**, **D**) to maximize the pattern correlation of precipitation between EOF1_R1 and the observed EOF1. The pattern correlation coefficients of precipitation between (**A**, **B**) and (**E**, **F**) are 0.81 and 0.60, respectively. (**G**, **H**) Rotated MJO EOF1 and EOF2 in (**C**, **D**) to maximize the pattern correlation of precipitation between EOF1_R2 and the EOF1 in Fig. 1**A**. The pattern correlation coefficients between (**G**, **H**) and Fig. 1(**A**, **B**) are 0.91 and 0.82, respectively.

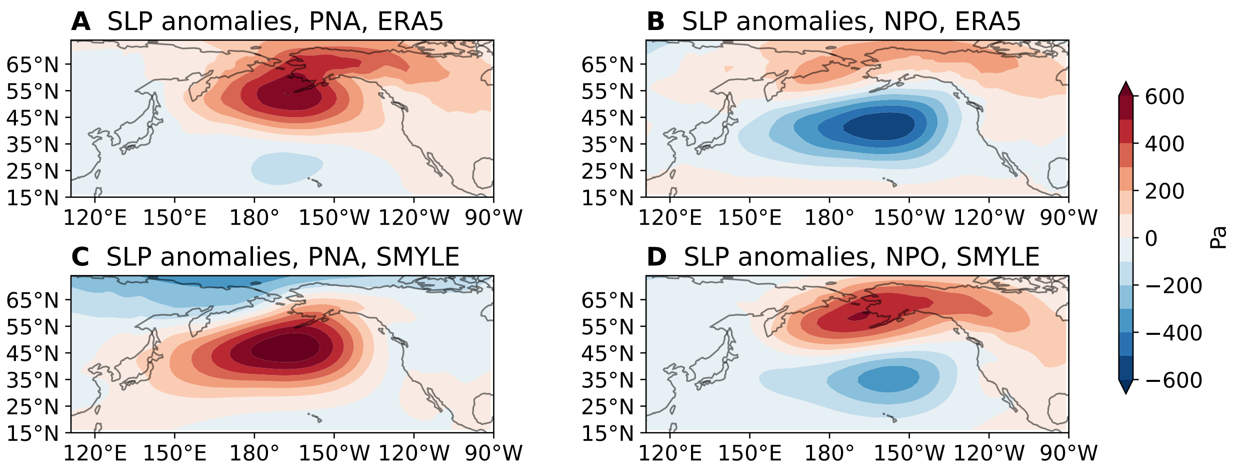

fig. S3. Sea level pressure anomalies associated with the PNA and NPO in the ERA5 reanalysis and SMYLE. (**A, B**) The first two Leading EOFs in ERA5. (**C, D**) The first two leading EOFs in SMYLE. The simulated PNA and NPO are displaced approximately 8° equatorward compared to the reanalysis, but their cyclonic circulations (indicated by blue shading) near Hawaii are weaker than those in the reanalysis.

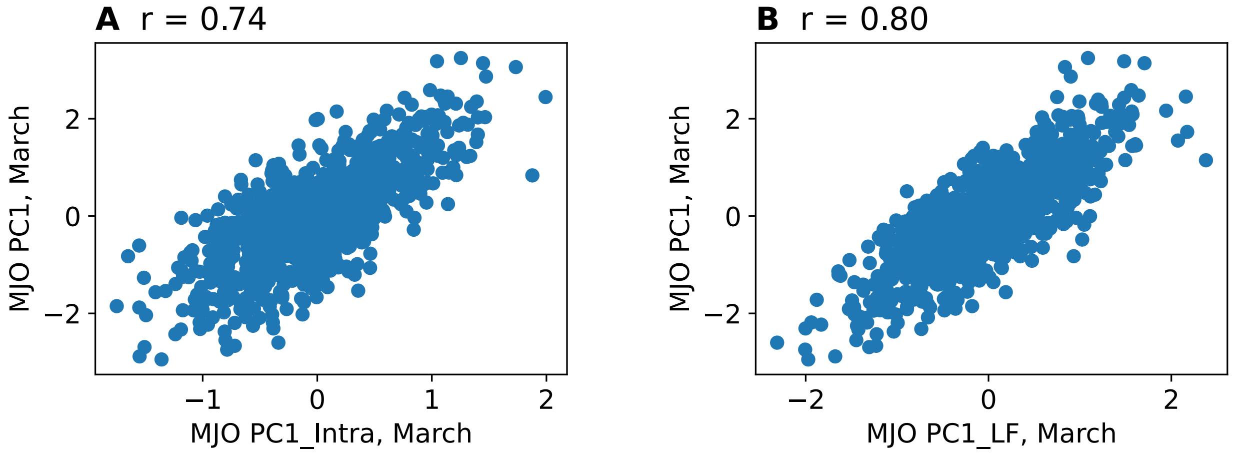

fig. S4. Relationships between MJO PC1 and its intraseasonal and low-frequency components. (**A**) Scatter plot of MJO PC1 versus its intraseasonal component, PC1_Intra. MJO PC1 is the principal component of EOF1 shown in Fig. 1A, and PC1_Intra is obtained by projecting the intraseasonal component of the March ensemble spread of precipitation onto the EOF1 precipitation pattern. (**B**) Scatter plot of MJO PC1 versus its low-frequency component, PC1_LF. PC1_LF is derived by projecting the low-frequency component of the March ensemble spread of precipitation onto the same EOF1 precipitation pattern.

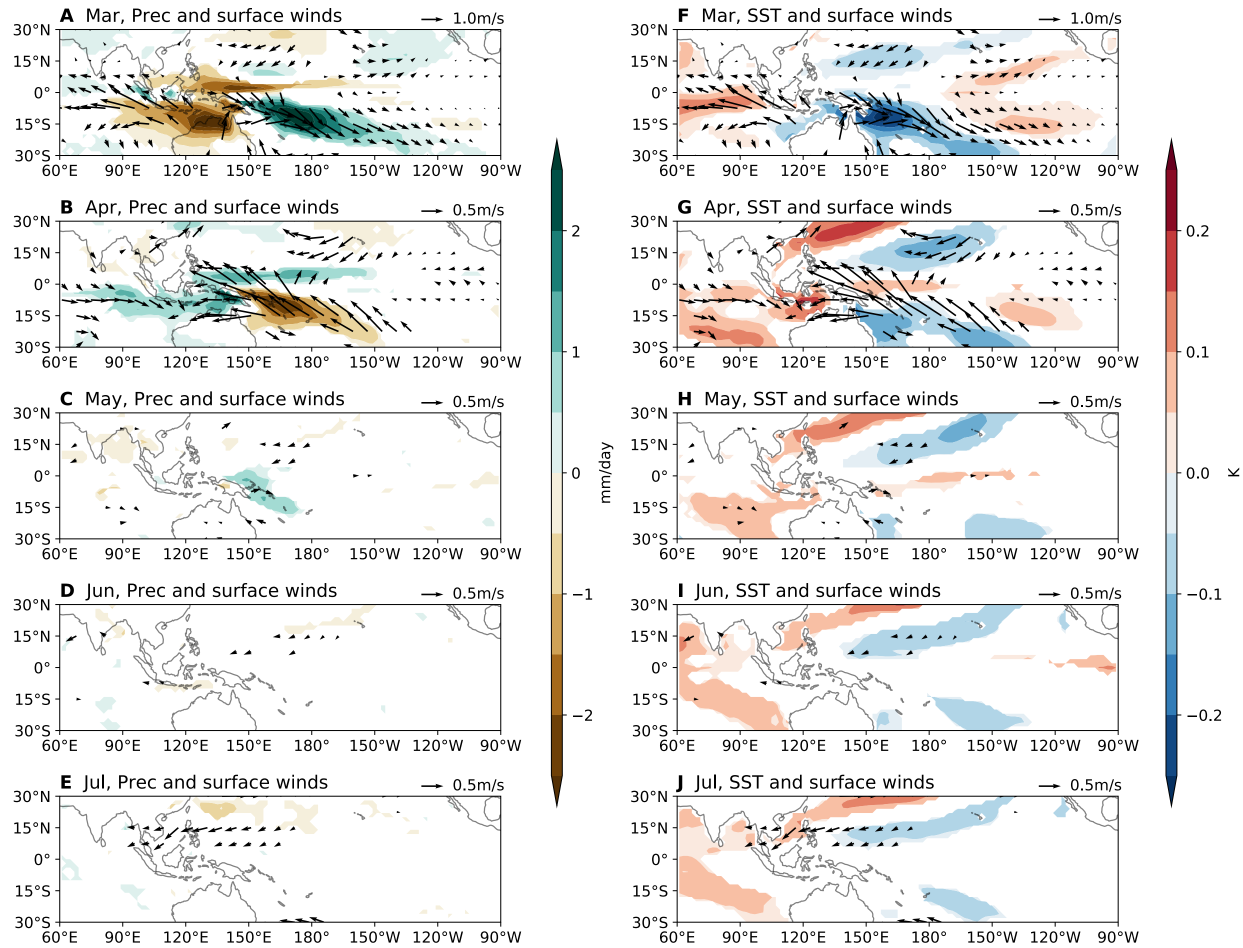

fig. S5. The ocean-atmosphere response to the March intraseasonal MJO. (A-E) Ensemble spread of precipitation and surface winds regressed onto PC1_Intra of the MJO (See Methods). **(F-J)** Same as the left panels, but with SSTs shown by color shading. Note that the scale for wind vectors differs in (**A**) and (**F**) from the other panels.

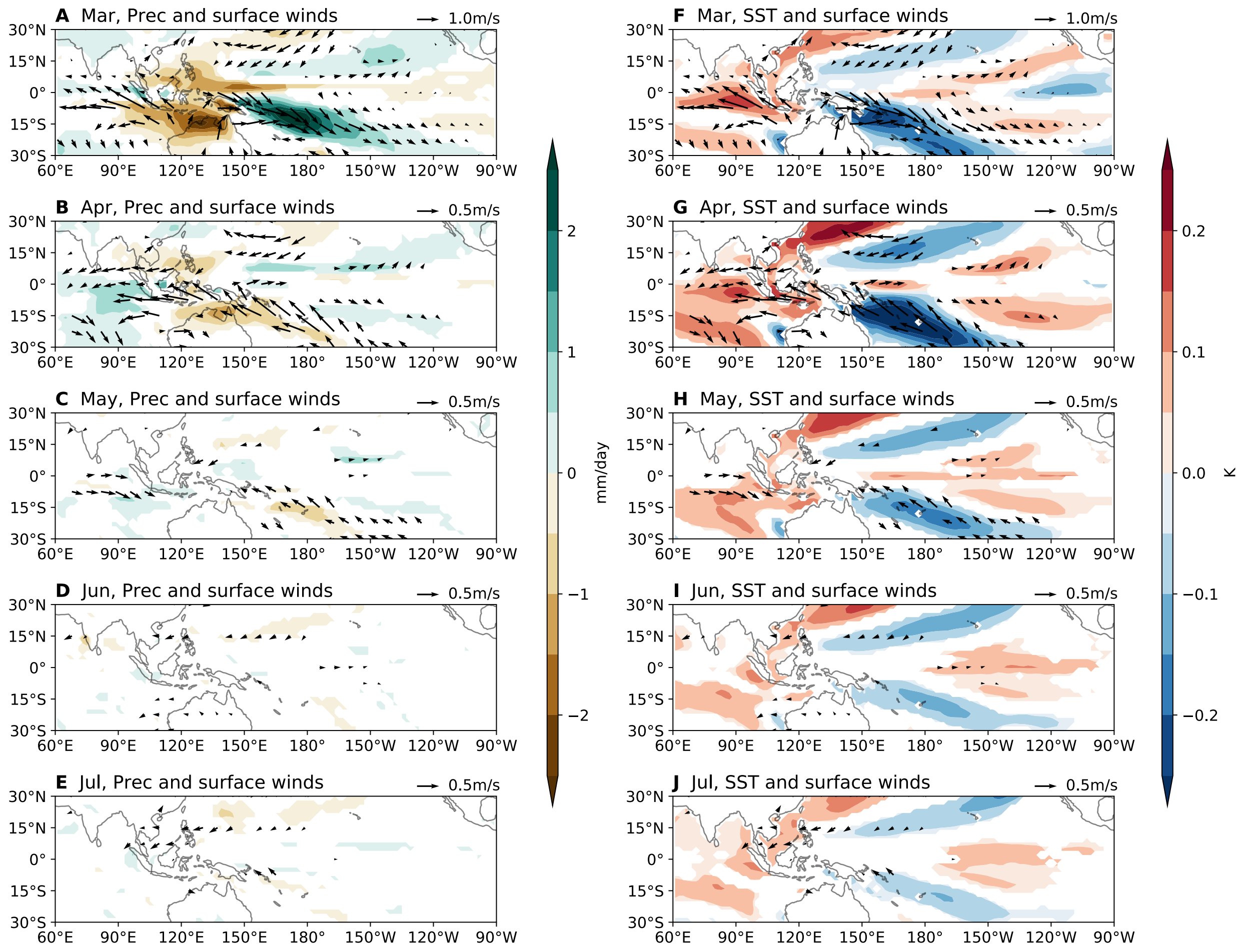

fig. S6. The ocean-atmosphere response to the March MJO-like low-frequency mode. (A-E) Ensemble spread of precipitation and surface winds regressed onto PC1_LF of the MJO (See Methods). **(F-J)** Same as the left panels, but with SSTs shown by color shading. Note that the scale for wind vectors differs in (**A**) and (**F**) from the other panels.

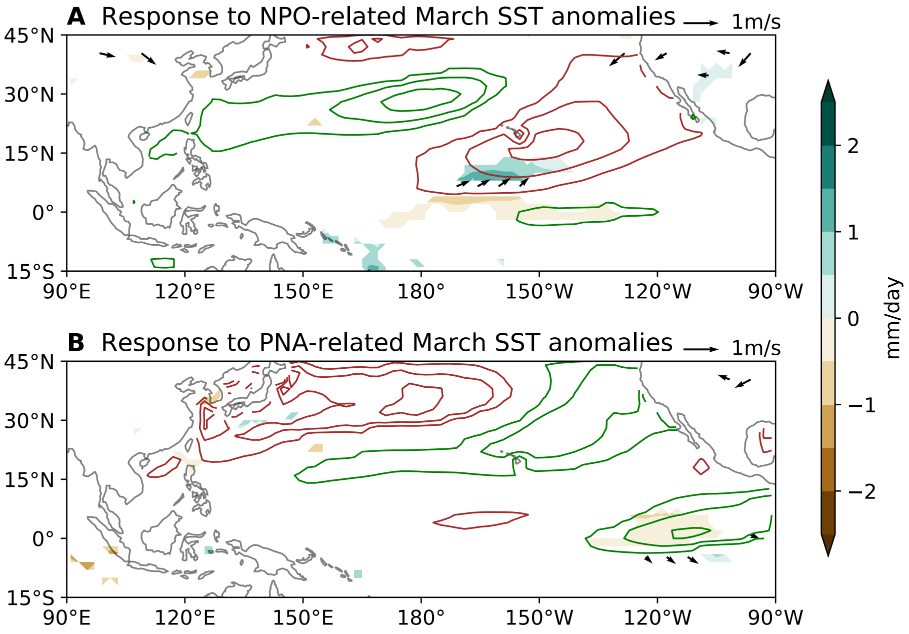

fig. S7. Atmospheric response to the NPO- and PNA-related SST anomalies in March. (A) Precipitation and wind response to the NPO-related SST anomalies shown in Fig. 5F. Brown and green contours represent positive and negative SST anomalies, respectively. (**B**) Same as (**A**), but for the PNA-related SST anomalies shown in Fig. 6F. Only the statistically significant response is shown.

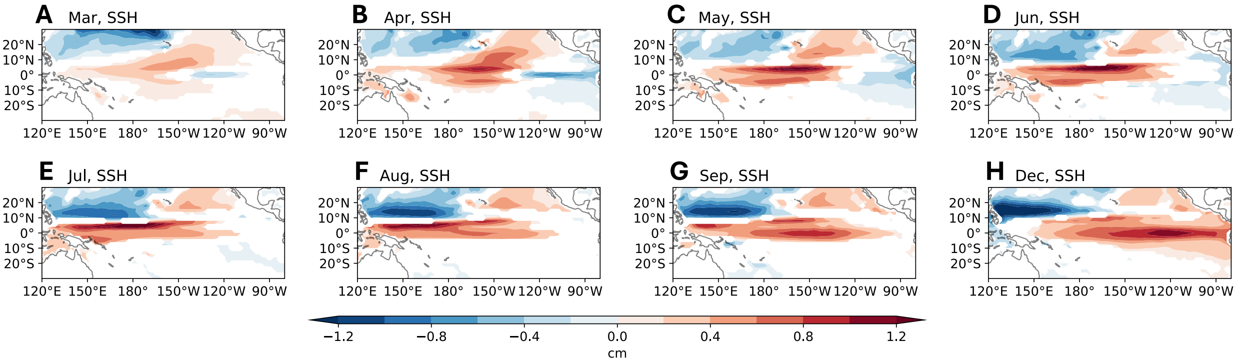

fig. S8. SSH response to the March NPO. (**A-H**) Ensemble spread of SSH regressed onto the PC2 of North Pacific atmospheric variability in March.

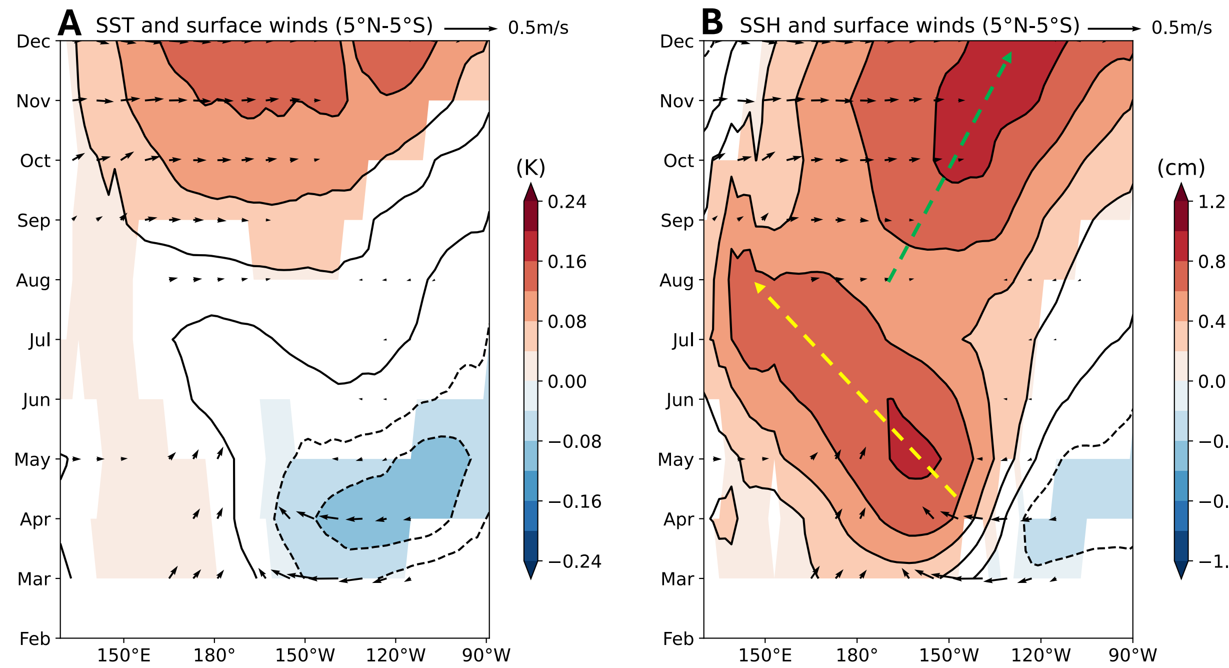

fig. S9. Equatorial oceanic and atmospheric response to the March NPO. (**A**) Ensemble spread of SST (contours) and surface wind vectors, averaged between 5°N-5°S, regressed onto the PC2 of North Pacific atmospheric variability in March. (**B**) Same as (**A**), but contours and color shading represent equatorially averaged sea surface height (SSH). Yellow and green arrows are used to indicate westward propagating oceanic Rossby waves and eastward propagating oceanic Kelvin waves, respectively.

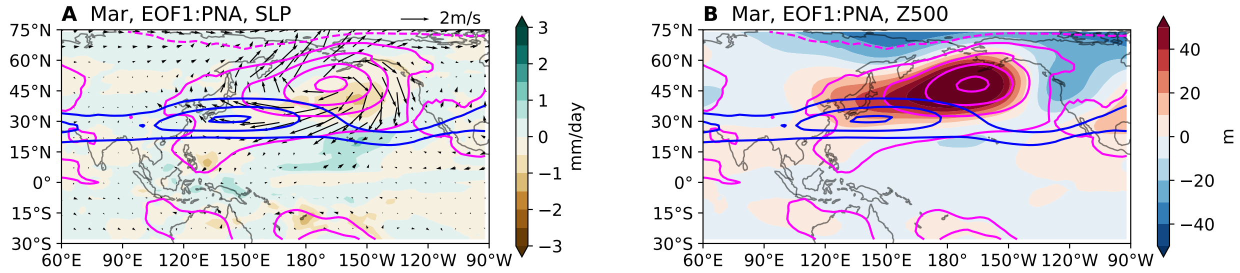

fig. S10. March PNA in the CAM6 control run forced with climatological monthly SST. EOF analysis is performed on the March sea level pressure (SLP) anomalies over the same domain as in Fig. 4. (**A**) EOF1 of SLP shown by magenta contours, with precipitation and surface winds regressed onto the corresponding PC. Blue contours indicate climatological zonal wind at 200hPa, highlighting the subtropical jet. (**B**) Geopotential height at 500hPa is regressed onto the PC of EOF1 in (**A**), illustrating the PNA quadrupole structure, including a cyclonic center near Hawaii that is often overlooked.

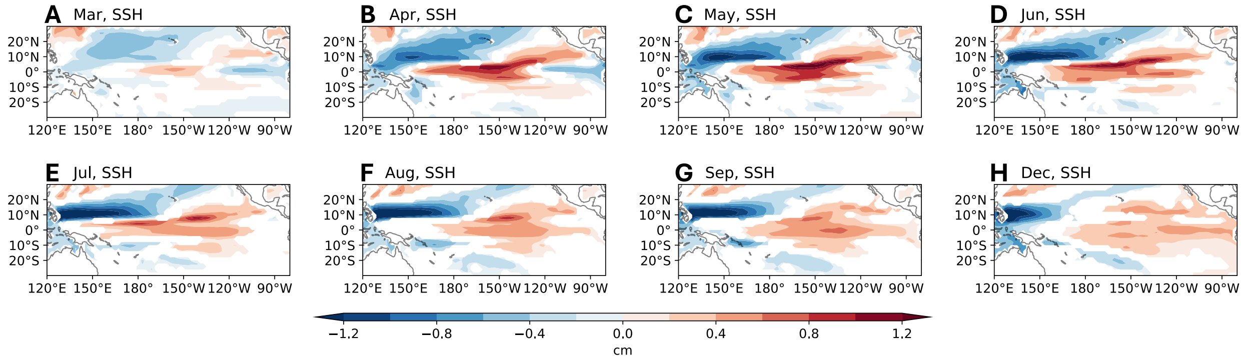

fig. S11. SSH response to the March Aleutian Low variability. (**A-H**) Ensemble spread of SSH is regressed onto the PC1 of North Pacific atmospheric variability in March.

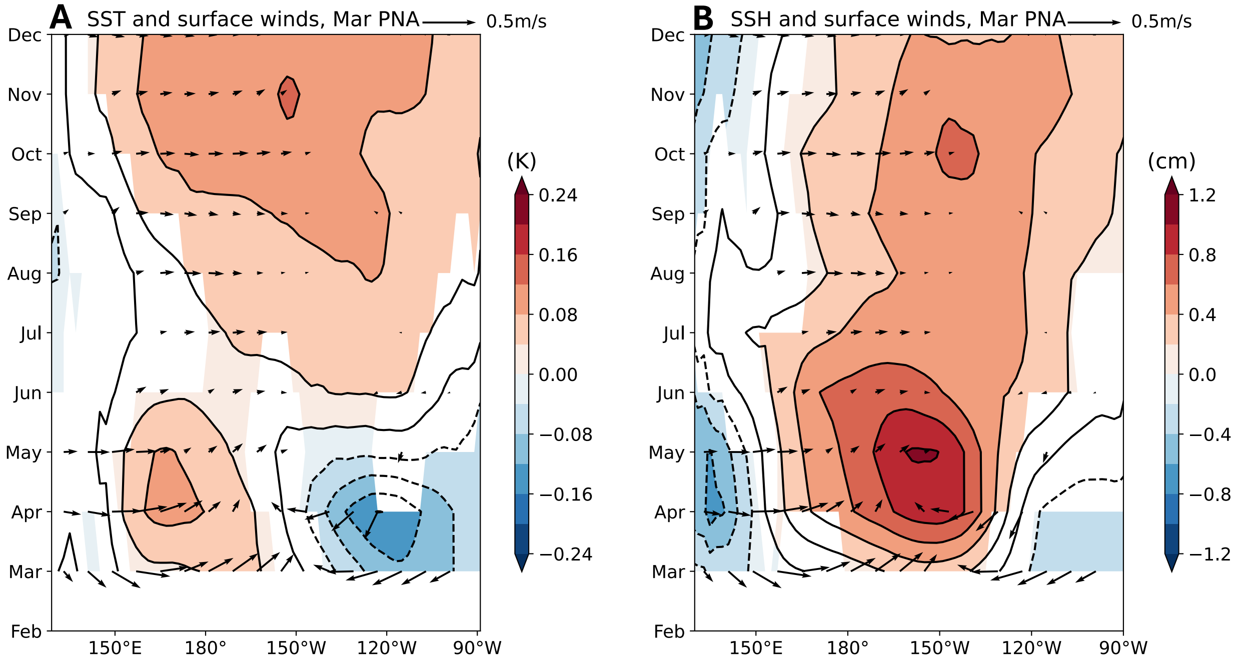

fig. S12. Equatorial oceanic and atmospheric response to the March Aleutian Low variability. (**A**) Ensemble spread of SST (contours) and surface wind vectors, averaged between 5°N-5°S, regressed onto the PC1 of North Pacific atmospheric variability in March. (**B**) Same as (**A**), but contours and color shading represent equatorially averaged sea surface height (SSH).

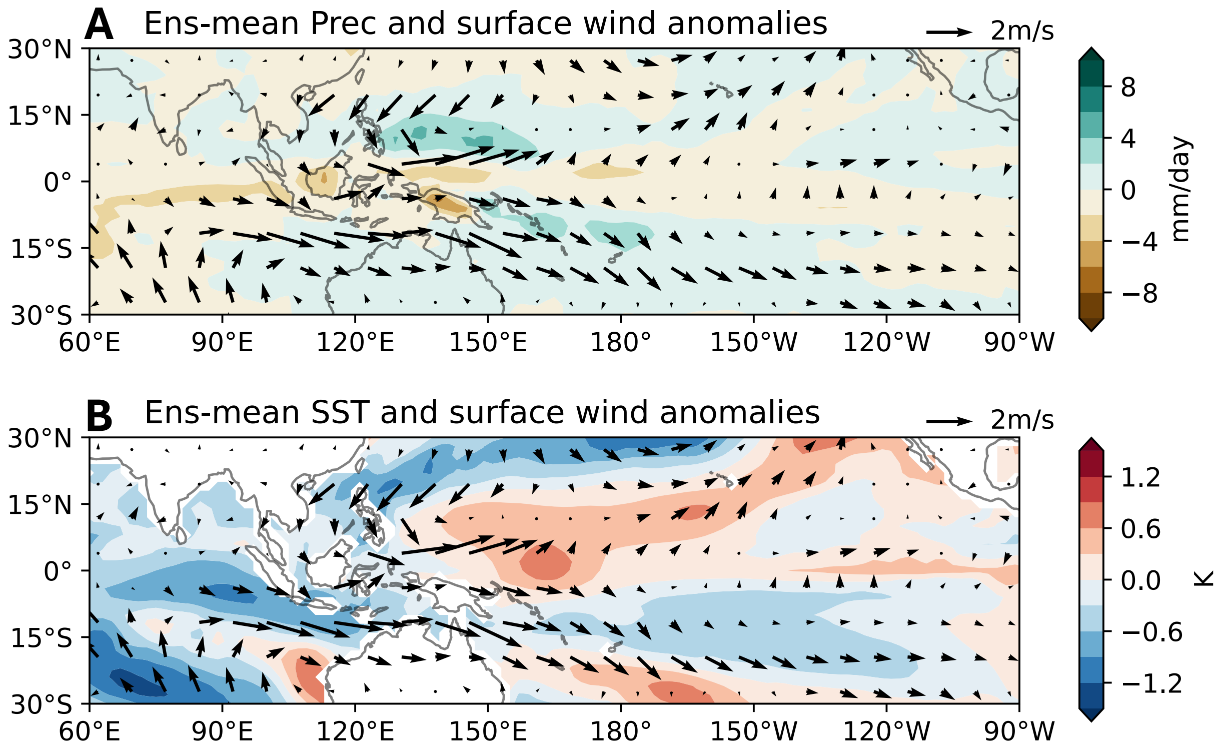

fig. S13. 50-member ensemble mean anomalies of atmospheric and oceanic conditions in March 1997. (**A**) precipitation (in color shading) and surface wind (black arrows) anomalies. (**B**) SST (in color shading) anomalies.

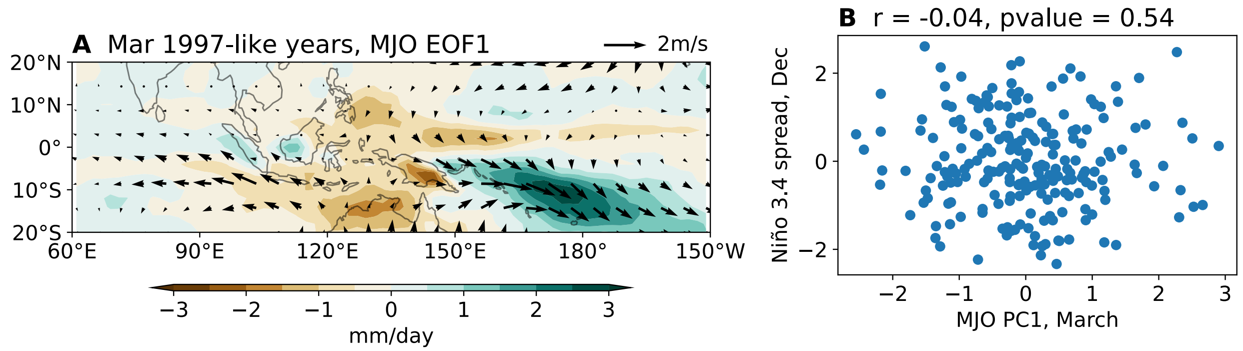

fig. S14: Simulated MJO in March of years resembling 1997, and its impacts on the following winter El Niño. (**A, B**) As in Fig. 8, except that we expand the ensemble sizes by including years in which the 20-member ensemble-mean SST anomalies exceed 0.3K in the western-central equatorial Pacific (150°E-170°W, 5°N-5°S) in March and 0.5K in the Niño 3.4 region in the following December. The MJO-related zonal wind anomalies averaged over the region 130°E-180°, 5°S-5°N are 0.36m/s, about 9% stronger than the average MJO in Fig. 1A.
